# Chitin Binding Proteins Act Synergistically with Chitinases in *Serratia proteamaculans* 568

**DOI:** 10.1371/journal.pone.0036714

**Published:** 2012-05-09

**Authors:** Pallinti Purushotham, P. V. Parvati Sai Arun, Jogadhenu S. S. Prakash, Appa Rao Podile

**Affiliations:** Department of Plant Sciences, School of Life Sciences, University of Hyderabad, Hyderabad, India; University of Hyderabad, India

## Abstract

Genome sequence of *Serratia proteamaculans* 568 revealed the presence of three family 33 chitin binding proteins (CBPs). The three *Sp* CBPs (*Sp* CBP21, *Sp* CBP28 and *Sp* CBP50) were heterologously expressed and purified. *Sp* CBP21 and *Sp* CBP50 showed binding preference to β-chitin, while *Sp* CBP28 did not bind to chitin and cellulose substrates. Both *Sp* CBP21 and *Sp* CBP50 were synergistic with four chitinases from *S. proteamaculans* 568 (*Sp* ChiA, *Sp* ChiB, *Sp* ChiC and *Sp* ChiD) in degradation of α- and β-chitin, especially in the presence of external electron donor (reduced glutathione). *Sp* ChiD benefited most from *Sp* CBP21 or *Sp* CBP50 on α-chitin, while *Sp* ChiB and *Sp* ChiD had major advantage with these *Sp* CBPs on β-chitin. Dose responsive studies indicated that both the *Sp* CBPs exhibit synergism ≥0.2 µM. The addition of both *Sp* CBP21 and *Sp* CBP50 in different ratios to a synergistic mixture did not significantly increase the activity. Highly conserved polar residues, important in binding and activity of CBP21 from *S. marcescens* (*Sm* CBP21), were present in *Sp* CBP21 and *Sp* CBP50, while *Sp* CBP28 had only one such polar residue. The inability of *Sp* CBP28 to bind to the test substrates could be attributed to the absence of important polar residues.

## Introduction

Chitin is a highly insoluble *β*-1, 4-liked polymer of *N*-acetylglucosamine (GlcNAc), and is the second most abundant polysaccharide (next only to cellulose) in nature. For the complete hydrolysis of chitin to GlcNAc, concerted action of chitinase (EC 3.2.1.14) and *β*-*N*-acetylglucosaminidase (EC 3.2.1.30) is essential. Chitin was extracted as two allomorphs, namely α- and β- forms [1]. The structures of α- and β forms of chitin differ only in the arrangement of piles of chains. Alternate chains are antiparallel in α-chitin, whereas they are all parallel in β-chitin. Among the chitin variants, α-chitin is the most abundant biopolymer in the nature. It occurs in fungal and yeast cell walls, krill, lobster and crab tendons and shells, and in shrimp shells, as well as in insect cuticle. Chitinase cleaves the glycosidic linkages between the adjacent GlcNAc residues to produce soluble oligosaccharides, which are further hydrolysed to GlcNAc by *β*-*N*-acetylglucosaminidases. Chitinase genes from bacteria have been cloned from both terrestrial and marine environments [2], [3]. Biochemical properties, catalytic mechanisms, and tertiary structures of chitinases were widely reported [4], [5]. A processive mechanism that improves substrate accessibility is generally considered favourable. But, it might in fact slow down enzymes. Improving substrate accessibility has been a key issue because this might reduce the need for using processive enzymes, which are intrinsically slow. Furthermore, carefully selected substrate-disrupting accessory proteins or domains might provide novel tools to improve substrate accessibility, and thus contribute to more efficient enzymatic processes [6].

Efficient chitin degradation also depends on the action of a family 33 chitin binding proteins (CBPs). The CBPs bind to the insoluble crystalline chitin, leading to structural changes and increased accessibility of substrate. The function of family 33 CBPs was first demonstrated for *Sm* CBP21 [7]. The details of CBPs and their binding preferences are given in Table 1. Studies of *Sm* CBP21 revealed that the protein has a “budded” fibronectin type 3-fold consisting of two β-sheets, arranged as a compact β-sheet sandwich-fold surface, having a at conserved region that binds chitin through interactions mediated mainly by polar amino acids [7], [8]. Conserved aromatic residues that have been suggested previously to play a role in chitin binding [9] were found in the interior of the protein, seemingly incapable of interacting with chitin. *Sm* CBP21 was designated as “chitin oxidohydrolase” as it acts on the surface of crystalline chitin, to introduce chain breaks and generates oxidized chain ends, promoting further degradation by chitinases [10]. Swapping of the chitin-binding domain in Bacillus chitinases improved the substrate binding affinity, and conformational stability [11].

**Table 1:** Details of bacterial CBPs and their binding preferences.

CBP Name	Source	Binding substrates	References
CBP21	*S. marcescens*	High preference to β-chitin followed by regenerated chitin and colloidal chitin	[15]
ChbB	*B. amyloliquefaciens*	Binds to both α- and β-chitin but shows preference to β-chitin	[17]
*Ll*CBP33A	*Lactococcus lactis*	Equally well to α- and β-chitin, followed by Avicel, colloidal chitin and chitin beads	[4]
CHB1	*St. olivaceoviridis*	Strictly to α- chitin	[25]
CHB2	*St. reticuli*	Strictly to α- chitin	[26]
CHB3	*St. coelicolor*	Most preferably to α -chitin followed by β -chitin	[27]
CbpD	*P. aeruginosa*	Colloidal chitin^a^	[28]
E7	*Thermobifida fusca*	Equally well to α- and β-chitin followed by bacterial microcrystalline cellulose	[29]
E8	*Thermobifida fusca*	Preferentially to β-chitin followed by α-chitin and microcrystalline cellulose	[29]
Cbp50	*B. thuringiensis*	Preferentially to β-chitin followed by α-chitin, colloidal chitin and cellulose	[18]
*Ef*CBM33A	*E. faecalis*	Binds both α- and β-chitin, but slightly more protein binds to β-chitin	[19]

a Details of binding to other substrates not available.

*S*. *proteamaculans* 568, a member of family Enterobacteriaceae, was isolated as a root endophyte from *Populus trichocarpa* [12]. According to the Carbohydrate Active enZyme data base (CAZy-http://www.cazy.org/) [13] *S*. *proteamaculans* 568 has at least eight genes involved in chitin turnover, coding for four family 18 chitinases (*Sp* ChiA, *Sp* ChiB, *Sp* ChiC and *Sp* ChiD), three family 33 CBPs (*Sp* CBP21, *Sp* CBP28 and *Sp* CBP50), and a family 20 *N*-acetylhexosaminidase (*Sp* CHB). The present study, describes the cloning and characterization of three CBPs from *S. proteamaculans* 568 and their synergy with *Sp* chitinases in degradation of natural chitin variants.

## Results

### Amplification and cloning of CBPs from *S. proteamaculans* 568

Three *Sp cbp* genes were amplified using gene specific primers with gDNA of *S. proteamaculans* 568 as template. The three *Sp* CBPs were predicted to contain N-terminal leader peptide directing *sec*-dependent secretion. Signal peptide was predicted using the SignalP server (http://www.cbs.dtu.dk/services/SignalP/). The genes were cloned without the signal peptide-encoding portion (*Sp cbp21*: 81 bp, *Sp cbp28*: 66 bp and *Sp cbp50*: 63 bp). The amplicons 0.51 kb of *Sp cbp21*, 0.76 kb of *Sp cbp28* and 1.35 kb of *Sp cbp50* were cloned in the *Nco* I and *Xho* I sites of pET 22b (+) and *Eco* RI and *Xho* I sites of pET- 28a (+), respectively.

The three *Sp* CBPs were over expressed with a C-terminal His-tag in *E. coli*. The expressed *Sp* CBPs were separated either from periplasmic fraction (*Sp* CBP21 and *Sp* CBP28) or from whole cell lysate (*Sp* CBP50) as soluble proteins, and purified using Ni-NTA agarose chromatography. The PelB signal sequence in pET-22 b (+) directs the expressed *Sp* CBP21 and *Sp* CBP28 proteins towards periplasmic space. SDS-PAGE analysis of the purified *Sp* CBPs revealed approximate molecular weight of 18.6, 28.0 and 50.0 kDa, which correspond to *Sp* CBP21, *Sp* CBP28 and *Sp* CBP50, respectively (Figure S1).

### Substrate binding preference of *Sp* CBPs

**Figure 1:**
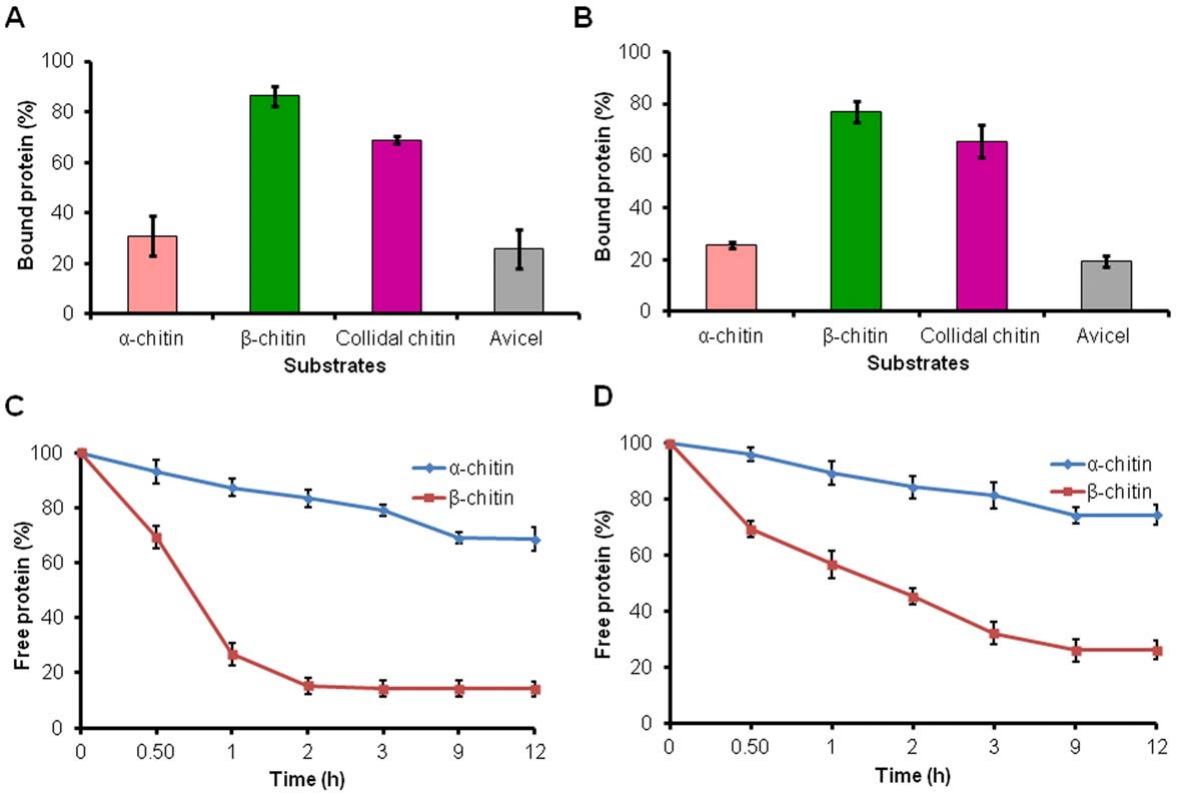
Binding of *Sp* CBPs to insoluble polymeric substrates. The reaction mixture (1 mL) containing 100 µg of *Sp* CBP21/*Sp* CBP50 and 1 mg of one of the insoluble substrates (α-chitin, β-chitin, colloidal chitin and Avicel) was incubated in 50 mM sodium phosphate buffer pH 7.0 under constant shaking at 1300 rpm at 37°C for 24 h. (A and B) The amount of bound protein was calculated as the difference in protein concentration before and after incubation with the insoluble substrates, (C and D) Decrease in free protein concentration after binding to α- and β-chitin was determined at different time points till 24 h. (A and C) *Sp* CBP21, (B and D) *Sp* CBP50. Vertical bars represent standard deviation of triplicate experiments.

The binding preference of *Sp* CBPs was assessed by incubating the protein with different insoluble polymeric substrates α-chitin, β-chitin and colloidal chitin and Avicel. The amount of *Sp* CBP bound to the respective substrate was analyzed by determining the protein concentration in the supernatant of the reaction mixture after 24 h of incubation. *Sp* CBP28 did not bind to any of the test substrates (data not shown). Both *Sp* CBP21 and *Sp* CBP50 bound equally high to β-chitin (86.2% and 77.0%), followed by colloidal chitin (68.8% and 65.6%), α-chitin (30.9% and 25.6%) and Avicel (25.9% and 19.3%) (Figures 1A and B).

The time course of binding was monitored for both the *Sp* CBPs as a function of time to find the time required for the *Sp* CBPs to get saturated with natural chitin variants. After separating the protein bound to chitin, the decrease in concentration of the unbound protein (remaining in the supernatant) was monitored at different time points up to 24 h. The binding of *Sp* CBP21 to β-chitin occurred rapidly and reached equilibrium within 6 h, while *Sp* CBP50 reached equilibrium by 12 h. On the other hand, *Sp* CBP21 and *Sp* CBP50 have established binding equilibrium to α-chitin by 12 h (Figures 1C and D).

**Figure 2:**
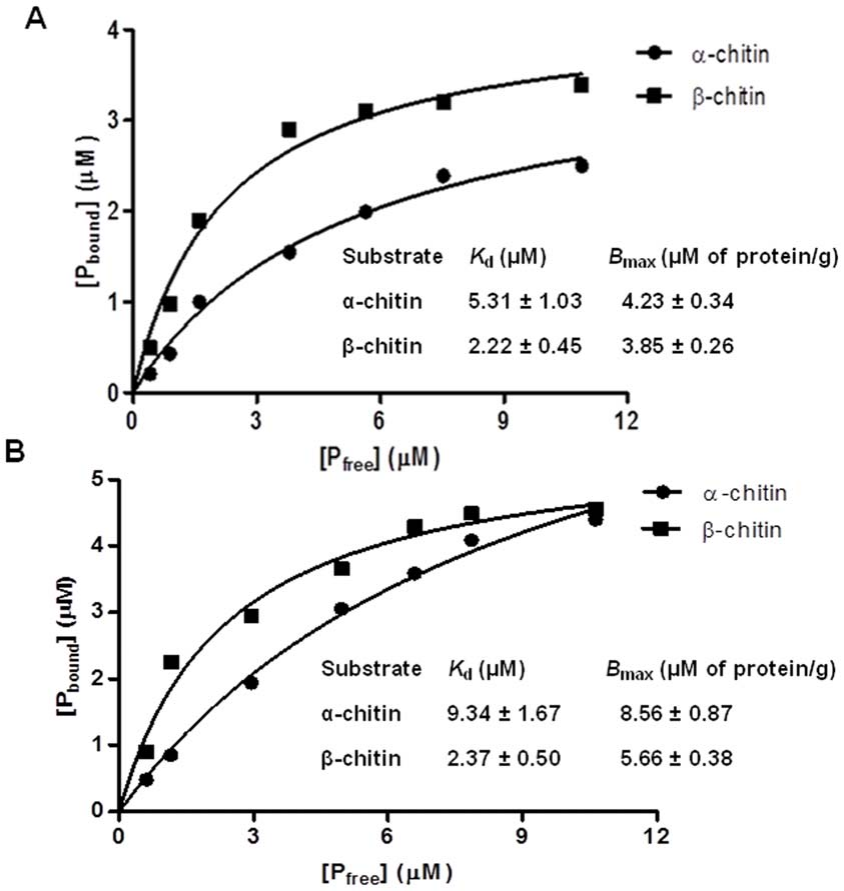
Equilibrium adsorption isotherms of *Sp* CBP21 and *Sp* CBP50 to α- and β-chitin. The reaction assay (1 mL) containing 1.0 mg substrates (α- and β- chitin) and varied concentrations of *Sp* CBP21 and *Sp* CBP50 starting from 0 to 10.0 µM was incubated (*Sp* CBP21 with α- and β-chitin, 12 h and 6 h, respectively; *Sp* CBP50 with α- and β-chitin, 12 h) at 37°C. The reaction mixtures were centrifuged and concentration of bound protein (P_bound_) and un-bound free protein (P_free_) was determined and plotted to fit into GraphPad Prism software version 5.0. All data sets were fitted to the equation for one-site binding by non-linear regression function, and to calculate *B*_max_ and *K*_d_ using GraphPad Prism software version 5.0. (A and B) The *K*_d_ and *B*_max_ values of *Sp* CBP21 and *Sp* CBP50 were shown in the inset table.

Adsorption isotherms of *Sp* CBP21 and *Sp* CBP50 towards α- and β-chitin were estimated and plotted with fixed concentration of substrate and varied concentrations of the CBPs. The dissociation binding constants (*K*_d_) of α- and β-chitin were estimated from the non-linear regression function. The *K*_d_ value of the *Sp* CBP21 to α-chitin (5.31±1.03 µM ) was much lower than the *K*_d_ value of *Sp* CBP50 to α-chitin (9.34±1.67 µM ), whereas the *K*_d_ value of *Sp* CBP21 to β-chitin (2.22±0.45 µM) was slightly lower than the *K*_d_ value of *Sp* CBP50 to β-chitin (2.37±0.5 µM) (Figure 2). Binding of *Sp* CBPs to the soluble substrates was also examined especially to investigate whether *Sp* CBP28 binds at least to soluble substrates. Electrophoretic mobility of *Sp* CBPs did not change in presence or absence of glycol chitin, CM cellulose and laminarin substrates (Figure S2).

### Homology modeling of *Sp* CBP21and *Sp* CBP50

**Figure 3:**
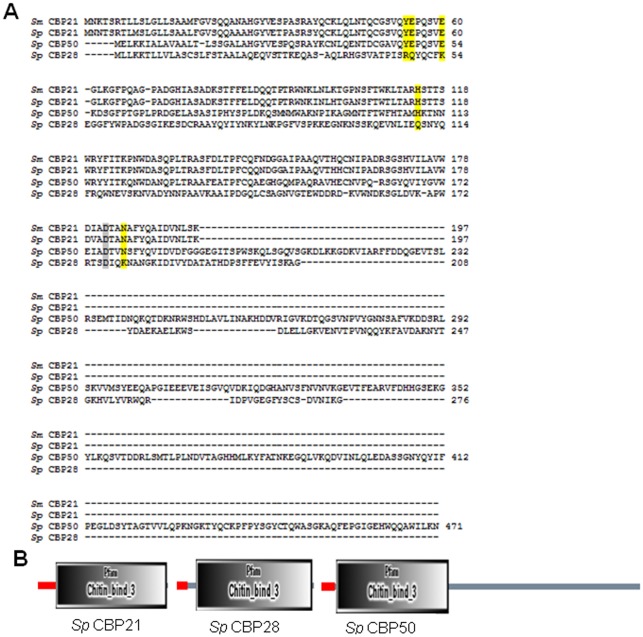
Sequence alignment and domain organisation for *Sp* CBPs. (A) Full-length sequences of *Sp* CBP21, *Sp* CBP28, *Sp* CBP50 and *Sm* CBP21 (CBP21 from *S. marcescens*) were aligned using clustalw2. Residues that are thought to be located in the binding surface for chitin present in *Sm* CBP21, *Sp* CBP21, *Sp* CBP50 and not present in *Sp* CBP28 are shaded in yellow (as derived from the crystal structure of *Sm* CBP21, as well as mutagenesis studies [7], [8]). Residue involved in the chitin-binding and functional properties of *Sm* CBP21 but also conserved in *Sp* CBP28 are shaded grey. The arrow indicates the terminal amino acid of the N-terminal signal sequence for respective CBPs. (B) The sequences of *Sp* CBPs were submitted to SMART domain data base (http://smart.embl-heidelberg.de/). The part indicated in red colour shows the signal peptide and the region Chitin_bind_3 indicates the chitin binding domain.

Sequence alignment of *Sp* CBP21, *Sp* CBP28 and *Sp* CBP50 with *Sm* CBP21 displayed 93%, 55% and 18% identity, respectively. The surface-located polar residues of *Sm* CBP21 [7], [8] were highly conserved in *Sp* CBP21 (Tyr-54, Glu-55, Glu-60, His-114, Asp-182, and Asn-185) and *Sp* CBP50 (Tyr-48, Glu-49, Glu-54, His-108, Asp-176, and Asn-179). *Sp* CBP28 had only one (Asp-176) matching residue (Figure 3A), out of six conserved polar residues. Both *Sp* CBP21 and *Sp* CBP50, showed similar β-chitin binding preference as shown by *Sm* CBP21, because of sequence homology and presence of conserved polar surface residues.

**Figure 4:**
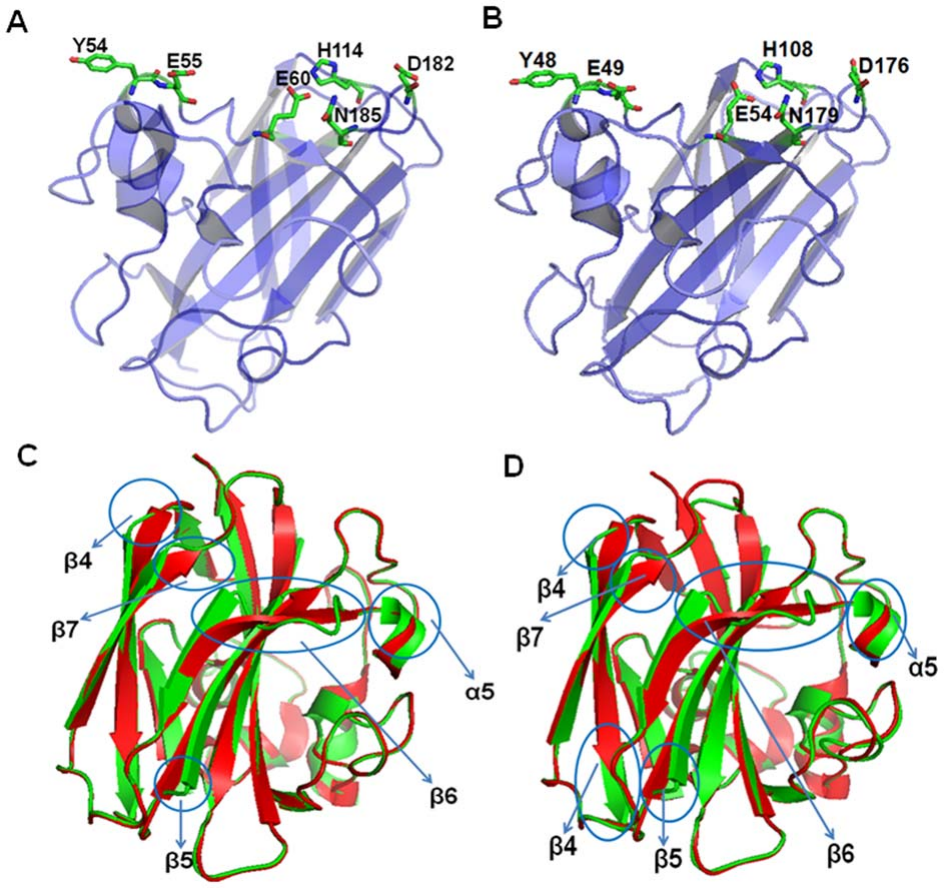
The 3D models of *Sp* CBP21 and *Sp* CBP50. (A and B) The models *Sp* CBP21 and ChBD region of *Sp* CBP50 were generated by Modeller9v8 (http://www.salilab.org/modeller/) using *Sm* CBP21 (PDB ID: 2BEM) as structure template. Residues important for chitin binding were shown in sticks representation with carbon, oxygen and nitrogen atoms colored light green, red and dark blue, respectively. The figures were prepared using PyMOL (http://www.pymol.org/), (C and D) Stereo view of the superimposed structure of *Sp* CBP21 and *Sp* CBP50 (green) with *Sm* CBP21 (red), respectively.

Sequence alignment and the SMART domain search data base revealed that the *Sp* CBP50 has N-terminal ChBD domain consisting of only 192 amino acids and the large part of the remaining sequence did not give any functional domain (Figure 3B). *Sp* CBP21 displayed high sequence identity to the sequences of the *Sm* CBP21 (93%). *Sp* CBP50 displayed only 55% of identity to *Sm* CBP21. The 3D structure models of *Sp* CBP21 and *Sp* CBP50 were generated using the template structure of *Sm* CBP21 (PDM ID: 2BEM) (Figures 4A and B). We have modeled only particularly ChBD region of the *Sp* CBP50 protein. The superimposition Cα atoms of the final model, on the template structure, gave a root mean square deviation (RMSD) of 0.182 Å and 0.164 Å for *Sp* CBP21 and *Sp* CBP50. Closer inspection of these structures revealed that the conformations of the several regions (α5, β4, β5, β6 and β7) were differing from the template structure (Figures 4C and D). Superimposition of *Sp* CBP21 and *Sp* CBP50 with the template revealed that the difference was mostly in the β-sheets.

### Synergism between *Sp* chitinases and *Sp* CBPs (*Sp* CBP21 & *Sp* CBP50) during degradation of α- and β-chitin

**Figure 5:**
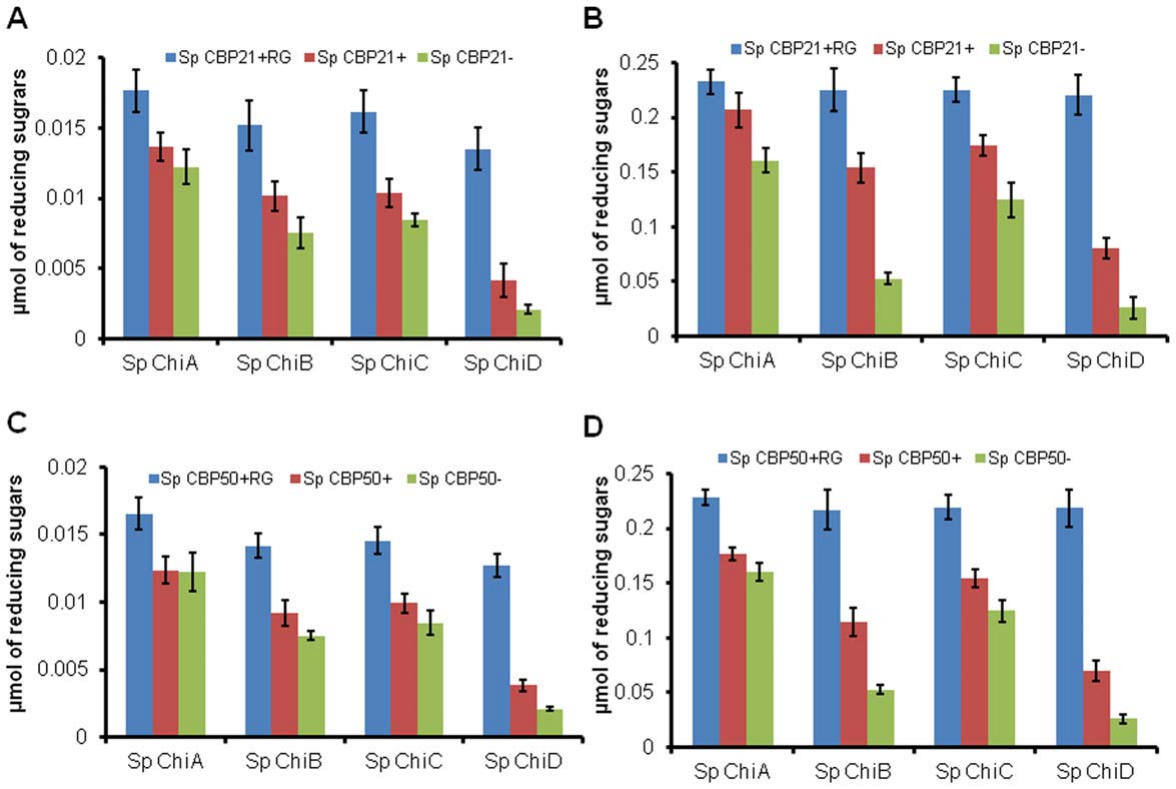
Degradation of α- and β-chitin by *Sp* chitinases in the absence or presence of *Sp* CBP21, *Sp* CBP50 and reduced glutathione. Reaction mixture (1 mL) containing 0.25 mg/mL of chitin substrates (α- and β-chitin), 1 µM of *Sp* chitinase (*Sp* ChiA/*Sp* ChiB/*Sp* ChiC/*Sp* ChiD) were incubated with 0.3 µM *Sp* CBP21 or *Sp* CBP50 and 1.0 mM reduced glutathione in 50 mM sodium phosphate buffer pH 7.0. After incubation at 37°C for 7 days at 1000 rpm, after every 24 h, 100 µL of reaction mixture was transferred. To this 100 µL of 0.02N NaOH was added to stop the reaction and stored at −20°C until products quantification by standard reducing end assay. Vertical bars represent standard deviation of triplicate experiments. (A and B) Degradation of α- and β-chitin by *Sp* chitinases in the presence/absence of *Sp* CBP21 and reduced glutathione (RG), (C and D) degradation of α- and β-chitin by *Sp* chitinases in the presence/absence of *Sp* CBP50 and reduced glutathione (RG). *Sp* CBP21+RG or *Sp* CBP50+RG: *Sp* CBP21/*Sp* CBP50 and reduced glutathione, *Sp* CBP21+ or *Sp* CBP50+: only *Sp* CBP21/*Sp* CBP50 without reduced gluthathione, *Sp* CBP21- or *Sp* CBP50 -: without *Sp* CBP21/*Sp* CBP50 and reduced glutathione.

The hydrolytic efficiency of four *Sp* chitinases (*Sp* ChiA, *Sp* ChiB, *Sp* ChiC, and *Sp* ChiD) on natural chitin variants was estimated in the presence/absence of *Sp* CBP21/*Sp* CBP50 and/or an external electron donor (reduced glutathione). The results indicate that, upon addition of *Sp* CBP21/*Sp* CBP50, efficiency of hydrolysis of α- and β-chitin by all the four *Sp* chitinases increased, especially in the presence of a reduced glutathione. Combination of both enzymes and the external electron donor led to total solubilization of β-chitin within 24 h, whereas only small fraction of the α-chitin was solubilised under the same conditions. The reactions shown in Figure 5 were sampled for up to one week following the 24-h time point. All reactions containing β-chitin reached complete solubilization after about one week of incubation, whereas none of the samples containing α-chitin were degraded completely after one week (results not shown). All three *Sp* CBPs showed optimum binding at 35°C-40°C, while all four *Sp* chitinases were also optimally active at the same temperature range. So, the synergistic experiments were carried out at 37°C (data not shown).

**Figure 6:**
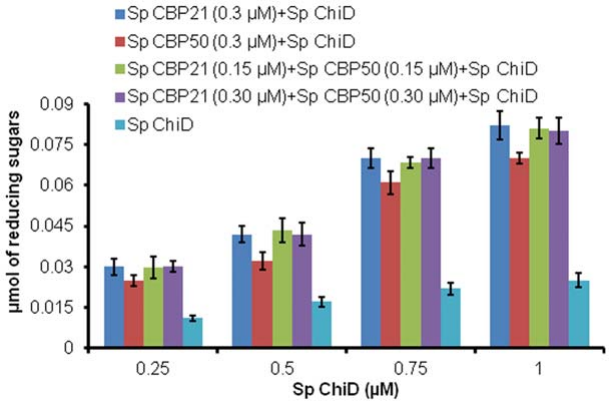
β-chitin hydrolysis enhancing effects of *Sp* CBP21 and *Sp* CBP50 with *Sp* ChiD. Reaction mixture (1 mL) containing 0.25 mg/mL of β-chitin, 0.25 µM/0.50 µM/0.75 µM/1.0 µM *Sp* ChiD incubated individually with 0.3 µM of *Sp* CBP21/*Sp* CBP50 or combining both *Sp* CBP21 and *Sp* CBP50 (0.15 µM +0.15 µM/0.30 µM +0.30 µM), in 50 mM sodium phosphate buffer pH 7.0. After incubation at 37°C for 24 h at 1000 rpm, 100 µL of reaction mixture was transferred. To this 100 µL of 0.02N NaOH was added to stop the reaction and stored at −20°C until products quantification by standard reducing end assay. Vertical bars represent standard deviation of triplicate experiments.

Figure 5 shows that the synergism exhibited by *Sp* CBP50 with *Sp* chitinases was lower when compared to the *Sp* CBP21 in degrading natural chitin variants. This was almost compensated when reduced glutathione was supplemented to the *Sp* CBP50. The product formation efficiency by *Sp* ChiA, *Sp* ChiB, *Sp* ChiC and *Sp* ChiD increased by 0.38, 1.04, 0.99 and 5.75-fold, respectively on α-chitin in presence of *Sp* CBP21 and reduced glutathione (Figure 5A). On the β-chitin substrate, the products formation increased by 0.44, 3.28, 1.20, and 7.50- folds in the presence of *Sp* CBP21 and reduced glutathione (Figure 5B). In the presence of *Sp* CBP50, *Sp* ChiA, *Sp* ChiB, *Sp* ChiC and *Sp* ChiD efficiency of α-chitin hydrolysis increased by 0.35, 0.88, 0.72 and 5.11-fold, respectively (Figure 5C) where as it was 0.42, 3.12, 0.76 and 7.43-fold higher, respectively on β-chitin (Figure 5D). The increased product formation in presence of *Sp* CBP21 and *Sp* CBP50 was relatively more with *Sp* ChiD on α-chitin compared to other *Sp* chitinases, while on β-chitin, *Sp* ChiD and *Sp* ChiB were having major advantage with both the *Sp* CBPs. Dose-response studies of the effect of *Sp* CBP21 and *Sp* CBP50 on *Sp* ChiD efficiency showed that *Sp* ChiD displayed maximum degradation rates at both the *Sp* CBP's concentrations ≥0.2 µM (Figure S3). The addition of both *Sp* CBP21 and *Sp* CBP50 in different ratios to a synergistic mixture of varied concentrations of *Sp* ChiD did not significantly increase the activity. *Sp* CBP21 appears to be compensating *Sp* CBP50 activity (Figure 6). Sampling of the synergistic mixtures (as above) at regular intervals, up to 24 h, also did not resulted improved product formation when compared to one time point sampling at 24 h (data not shown).

## Discussion

The ChBMs (chitin binding modules) are known to occur as discrete domains in chitinases and also exist independently as CBPs grouped in families 14, 18, and 33. Families 14 and 18 constitute small anti-fungal proteins that share a structurally similar chitin-binding motif [14]. Family 33 CBPs are mainly found in bacteria and viruses. Bacterial family 33 CBPs are expressed and secreted during chitin degradation. *Sm* CBP21 invades the chitin matrix to dissolve individual polymers, and make them more accessible to degradation by chitinases [8].

Analysis of genome sequence of *S. proteamaculans* 568 revealed the presence of genes coding for three CBPs of family 33. The *Sp* CBP21 was designated according to its homology to the reported *Sm* CBP21. *Sp* CBP21 showed high sequence identity to *Sm* CBP21 (93%). There were no reports on the presence of additional CBPs in *S. marcescens*. The two additional CBPs of *S. proteamaculans* were designated as *Sp* CBP28 and *Sp* CBP50 according to the estimated molecular weights (without the signal peptide) of these two proteins. *S. proteamaculans* 568 codes for at least 3 CBPs, while others produce either one or two CBPs (Table 1). The three *Sp* CBPs were arranged distantly in the genome of *S. proteamaculans* 568 and shares conserve 8 bp regions (5′-C(C/T/A) C(C/T) (T/G) G (C/A) (C/G)-3′) in the upstream sequences with other Sp chitinolytic genes (data not shown). Therefore, these *Sp cbp* genes might be co-ordinately controlled by the same regulatory protein(s) along with other *Sp* chitinolytic genes. Since *S. proteamaculans* 568 produced additional CBPs, characterization of CBPs in terms of their binding properties as well as synergism with *Sp* chitinases in chitin degradation was investigated.

The amino acid sequence of Sp CBP21 was BLAST at NCBI database to search for homologs. The result displayed 93% identity to Sm CBP21 (BAA31569), 57% to CBP from Bacillus cereus G9241 (EAL13960) and CBP from B. thuringiensis serovar tochigiensis BGSC 4Y1, 44% to Cbp21 from from B. anthracis str. CDC 684 (ACP12567), 41% to CHB2 from Streptomyces reticuli (EEM22267), 30% to CbpD from Stenotrophomonas sp. SKA14 (EED38588) and 27% to CbpD from Pseudomonas aeruginosa (AF196565). Sp CBP21 contained a signal peptidase site between amino acid residues Ala-27 and His-28. ChBD (Chitin binding domain) was present from His-28 through Asn-194.

Sp CBP28 showed 36% identity to CBP1 from Pseudoalteromonas piscicida (BAB79619) and CBP from Vibrio cholerae NCTC 8457 (EAZ71564), 33% to CBP from B. thuringiensis BMB171 (ADH07314) and CBP from B. cereus ATCC 1457 (AAP09751), and 31% to CBP from Streptomyces sp. e14 (EFF90245). A signal peptidase site was located between amino acid residues Ala-22 and Gln-23 of Sp CBP28. ChBD was present from His-39 through Asn-273.

The BLAST search for Sp CBP50 homologs displayed 62% identity to N-acetylglucosamine-binding protein A from Enterobacter cloacae subsp. cloacae ATCC 13047 (ADF60226), 57% to CBP21 from S. marcescens (BAA31569), 46% to CBP from Shewanella sp. HN-41 (ABK37291), 45% to CBP from Aeromonas hydrophila subsp. hydrophila ATCC 7966 (ABK37291), 40% to CHB2 from Streptomyces reticuli (CAA74695), and 24% to CbpD from Vibrio harveyi HY01 (EDL70242). Sp CBP50 contained a signal peptidase site between amino acid residues Ala-21 and His-22 and ChBD present from His-22 through Asp-188.

Binding studies of *Sp* CBPs to chitin variants and cellulosic substrates revealed that *Sp* CBP28 did not to bind to the test substrates. *Sp* CBP21 and *Sp* CBP50 were similar to the *Sm* CBP21, with maximum binding to β-chitin followed by α-chitin, colloidal chitin and Avicel (Figures 1A and B). As α-chitin has strong intersheet and intrasheet hydrogen bonding, compared to weak hydrogen bonding in intrasheets of β-chitin, the *Sp* CBP21 and *Sp* CBP50 preferably bound to β-chitin.

The difference in substrate preference was mainly attributed to the difference in the amino acid sequence of respective CBPs. The only available three-dimensional structure of close to *Sp* CBPs was *Sm* CBP21, which binds exclusively to β-chitin [15]. The combination of sequence and structural information with the results of site-directed mutagenesis showed that the surface of family 33 CBPs contains a patch of highly conserved, mostly polar residues (Tyr54, Glu55, Glu60, His114, Asp182, and Asn185), important for binding to chitin, and also for a positive effect on the efficiency of chitinase [8]. *Ll*CBP33A which binds equally to α- and β-chitin had two substitutions in the conserved surface patch. Both these residues were known to be important for *Sm* CBP21 functionality [8]. Ser63 occurs at a position at which *Sm* CBP21 had a tyrosine (Tyr54), and other family 33 CBPs have tryptophan e.g. Trp57 in CHB1 from *St. olivaceoviridis*, which has been shown to be important for the ability of CHB1 to bind α-chitin [16]. Asn64 occurs instead of a Glu55 of *Sm* CBP21. The closest homologue of *Ll*CBP33A is ChbB from *B. amyloliquefaciens* (66% sequence identity), which binds both α- and β-chitin [17]. ChbB differs from *Sm* CBP21 in the same two positions as *Ll*CBP33A: Tyr54 is replaced by Asp62, and Glu55 is replaced by Asn63.

Alignment of the amino acid sequence of *Sp* CBPs with *Sm* CBP21revealed that all the amino acid residues that are important in chitin binding [7], [8] are conserved in *Sp* CBP21 (Tyr-54, Glu-55, Glu-60, His-114, Asp-182, and Asn-185) and *Sp* CBP50 (Tyr-48, Glu-49, Glu-54, His-108, Asp-176, and Asn-179), while *Sp* CBP28 showed only one conserved residue (Asp-176) (Figure 3A). Minimum homology and absence of important polar residues could be the reason for the inability of *Sp* CBP28 to bind to substrates. It remains to be confirmed whether *Sp* CBP28 has a role other than chitin binding. The presence or absence of conserved amino acid residues in CBPs, therefore, conferred substrate binding preference.

The binding of *Sp* CBP21 and *Sp* CBP50 to β-chitin occurred rapidly and reached equilibrium within 6 and 12 h, respectively. *Sm* CBP21 established binding equilibrium after 16 h of incubation [7]. *Sp* CBP21 and *Sp* CBP50 showed relatively slow binding to α-chitin and reached equilibrium by 12 h. *Ll*CBP33A from *Lactococcus lactis* subsp. *lactis* established binding equilibrium by approximately 24 h of incubation with both α- and β-chitin [4]. In agreement with the binding assay, the lower *K*_d_ values of *Sp* CBP21 and *Sp* CBP50 indicate that both these CBPs have high binding strength towards the β-chitin in comparison with α-chitin. *Sp* CBP21 and *Sp* CBP50, *K*_d_ values towards the β-chitin were relatively higher, while *B*_max_ values were lower when compared to the *K*_d_ and *B*_max_ values of reported CBPs from *S. marcescens* and *B. thuringiensis* serovar *konkukian* [7], [18]. None of the *Sp* CBPs bound to soluble substrates, as observed for *Sm* CBP21 by Vaaje-Kolstad et al., [7].

*Sm* CBP21 catalyzes cleavage of glycosidic bonds in crystalline chitin [10], opening up the inaccessible polysaccharide material for hydrolysis by normal glycoside hydrolases. Such unique enzymatic activity was discovered after detection of traces of previously unidentified chitooligosaccharides up on incubation of β-chitin nano whiskers with *Sm* CBP21. Vaaje-Kolstad et al., [7] reported that the CBPs bind to the insoluble crystalline substrate, leading to both structural changes and increased substrate accessibility to the *Sm* chitinases (*Sm* ChiA, *Sm* ChiB and *Sm* ChiC). The *Sm* CBP21 strongly promoted hydrolysis of crystalline β-chitin by *Sm* ChiA and *Sm* ChiC, while *Sm* ChiB it was essential for complete degradation, and *Sm* CBP21 activity was boosted by external electron donor [10]. Vaaje-Kolstad et al., [4], [19] also showed that the *Ll*CBP33A and *Ef*CBM33A increased the hydrolytic efficiency of *Ll*Chi18A and *Ef*Chi18A, respectively to both α- and β-chitin. These results show the general importance of CBPs in chitin turnover.

Among the four *Sp* chitinases, *Sp* ChiA, *Sp* ChiB and *Sp* ChiC released chitobiose as major end product [20], while *Sp* ChiD released GlcNAc from chitin substrates (based on HPLC) (data not shown). Hydrolysis of natural chitin variants by four *Sp* chitinases in the presence of *Sp* CBP21 and *Sp* CBP50 showed that efficiency of all the four *Sp* chitinases increased with both α- and β-chitin (Figure 5). The *Sp* chitinases were less active on α-chitin (data not shown) and *Sp* CBP21 and *Sp* CBP50 had only minor binding preference to α- chitin. Therefore, there was no significant increase in the product formation on α-chitin. The addition of *Sp* CBP21 and *Sp* CBP50 had only minor effect on hydrolysis of β-chitin by *Sp* ChiA and *Sp* ChiC, while efficiency of *Sp* ChiB and *Sp* ChiD increased significantly high. These results are in line with the earlier report on *Sm* ChiB [8] that was dependant on *Sm* CBP21 in β-chitin degradation. Interestingly, in both the organisms, the *cbp21* gene is located 1.5 kb downstream to the *Sm chiB*. It was even reported that *Sm* CBP21 was produced along with three chitinases, *Sm* ChiA, *Sm* ChiB and *Sm* ChiC [15]. Overall, with both the chitin substrates (α- and β-chitin) *Sp* ChiD obtained major benefit from *Sp* CBP21 and *Sp* CBP50. *Sp* ChiD is a single GH18 domain enzyme that exhibited lower activity on both α- and β-chitin when compared to other *Sp* chitinases. CBP-mediated enhancement of substrate availability increased the efficiency of *Sp* ChiD. *Sp* CBP21 and *Sp* CBP50 are useful to convert the chitin biomass into production of chitooligosaccharides, which are useful in agriculture, food, and pharmaceutical industries. The uniqueness of *Sp* CBP28 is being investigated in terms of its importance in biology of *S. proteamaculans* 568.

## Materials and Methods

### Chemicals and enzymes

Restriction enzymes, T4 DNA ligase, and *Pfu* DNA polymerase were from MBI Fermentas (Ontario, Canada). Primers were procured from Eurofins India (Bangalore, India). Isopropyl-β-D-thiogalactoside (IPTG), ampicillin, kanamycin, chloramphenicol and all other chemicals were purchased either from Sigma–Aldrich (Missouri, USA), or Merck (Darmstadt, Germany), or Hi-media labs (Mumbai, India). The polymeric substrates α- and β-chitin were kindly provided by Mahtani Chitosan (Veraval, India). Colloidal chitin (CC) was prepared according to Berger and Reynolds [21].

### Bacterial strains, plasmids, and media

*Serratia proteamaculans* 568 was grown in Luria-Bertani (LB) broth at 28°C for 16 h for the extraction of gDNA (QIAgen, Duesseldorf, Germany). Plasmid vectors pET 22b(+), pET 28a(+), and *Escherichia coli* Rosetta-gami 2(DE3) were used as vector and host (Novagen, Darmstadt, Germany) for expression, respectively. To express CBP genes, *E. coli* Rosetta-gami 2(DE3) carrying pET 22b(+) or pET 28a(+) was grown in LB broth with ampicillin (100 µg/mL) and chloramphenicol (25 µg/mL) or kanamycin (50 µg/mL) and chloramphenicol (25 µg/mL), respectively.

### Amplification and cloning of *Sp* CBPs

Three genes encoding CBPs (*Sp cbp21*, *Sp cbp28* and *Sp cbp50*; GenBank accession no. ABV42576.1, ABV42205.1, and ABV43333.1, respectively) were amplified from the gDNA by referring to the annotated sequence of *S. proteamaculans* 568 at 55°C annealing temperature using gene specific forward and reverse primers listed in Table 2. Expression vectors, and the amplicons were separately digested with *Nco* I and *Xho* I (pET 22b(+), *Sp cbp21* and *Sp cbp28*), and *Eco* RI and *Xho* I (pET 28a(+) and *Sp cbp50*), gel purified and ligated using T4 DNA ligase at 16°C for 16 h. The resultant plasmids were designated as pET 22b–*Sp cbp21*, pET 22b–*Sp cbp28* and pET 28a–*Sp cbp50* to express *Sp* CBP21, *Sp* CBP28 and *Sp* CBP50, respectively in *E. coli*.

**Table 2:** List of primers used for the amplification of genes coding for chitin binding proteins and chitinases from *Serratia proteamaculans* 568.

Gene I.D	Gene	Primer	Primer sequences^a^ (5′<$>\vskip -1\scale 40%\raster="rg1"<$>3′)	Restriction site
ABV42576.1	*Sp cbp21*	Forward	AAT AAC CAT GGT TCA CGG CTA TGT CGA AAC	*Nco* I
		Reverse	GCA TAC TCG AGT TTA GTC AAA TTA ACG TC	*Xho* I
ABV42205.1	*Sp cbp28*	Forward	ATA CAC CAT GGT TCA GGA GCA AGT TTC CAC TAC	*Nco* I
		Reverse	AAT CAC TCG AGG CCT TTG ATA TTG ACG TCA C	*Xho* I
ABV43333.1	*Sp cbp50*	Forward	TCA AGA ATT CCA TGG TTA TGT TGA ATC GCC G	*Eco* RI
		Reverse	ATA AAC TCG AGT CAG TTT TTT AAT ATC CAG GCT TGT TGC C	*Xho* I
ABV39247.1	*Sp chiA*	Forward	CAA TAA CCA TGG CCG TAC CGG GTA AGC CTA C	*Nco* I
		Reverse	AAT AAC TCG AGT TGC GTG CCG GCG CTG TTG	*Xho* I
ABV40327.1	*Sp chiB*	Forward	CAT AAC CAT GGT GTC CGA ACG TAA AGC CGT TAT TG	*Nco* I
		Reverse	AAT AAC TCG AGT GCC ACG CGG CCC ACT TTC	*Xho* I
ABV42574.1	*Sp chiC*	Forward	GCT CAC CAT GGT GAG CAC CAA TAA TAT TAT CAA TGC	*Nco* I
		Reverse	AAT AAC TCG AGG GCG GTC AAC TGC CAC AG	*Xho* I
ABV41826.1	*Sp chiD*	Forward	TAA TAC CAT GGG TGC CGG CAT GGC TCA TG	*Nco* I
		Reverse	AAT AAC TCG AGC TGT TTC CCG TTA ATC C	*Xho* I

a Sequences underlined represent restriction sites.

### Expression and purification of *Sp* CBPs

Expression and purification of *Sp* CBP21, *Sp* CBP50 and *Sp* CBP28 were done as described by Neeraja et al., [22], except that the *Sp* CBP50 was isolated from whole cell lysate by sonicating the cell pellet. The cell pellet was suspended in Ni-NTA equilibration buffer (50 mM NaH_2_PO_4,_ 100 mM NaCl and 10 mM imidazole pH 8.0). The cells were lysed by sonication at 20% amplitude with 30×15 s pulses (with 20 s delay between pulses) on ice, with a Vibra cell Ultrasonic Processor, converter model CV33, equipped with a 3 mm probe (Sonics, Newtown, CT, USA). To pellet the insoluble cell debris, sonicate was centrifuged at 15,200× *g* for 10 min at 4°C. The expressed protein was purified using Ni-NTA column as the expressed protein having C-terminal His-tag. After purification, the *Sp* CBPs were buffer exchanged with 50 mM sodium phosphate buffer pH 7.0 using Macrosep Centrifugal Devices (Pall Corporation, USA), and stored at 4°C until use.

### Protein measurement

Purified *Sp* CBPs were quantified by BCA (bicinchonic acid) protein assay kit (Novagen, USA) using a standard calibration curve constructed from BSA (bovine serum albumin). For the chitin binding assay, protein concentration was measured from the absorption at 280 nm using the molar extinction coefficients (ε) calculated from the amino acid composition of the protein as described by Pace et al., [23].

### Insoluble substrate binding specificity

Insoluble substrate binding of *Sp* CBPs was done as described by Vaaje-Kolstad et al., [7] with slight modifications. The substrates, α-chitin, β-chitin, colloidal chitin and Avicel were used as insoluble substrates, and BSA was used as a background control for nonspecific adsorption. The binding mixture (1 mL) was incubated for 24 h at 37°C with vigorous shaking at 1300 rpm on thermomixer (Thermomixer comfort; Eppendorf, Hamburg, Germany).

### Time course binding of *Sp* CBP21 and *Sp* CBP50 towards α- and β-chitin

To study the time at which the binding of *Sp* CBP21 and *Sp* CBP50 (described above) was getting saturated with natural chitin variants (α- and β-chitin) was assessed at different time points up to 24 h.

### Adsorption isotherms of *Sp* CBP21 and *Sp* CBP50 towards α- and β-chitin

Adsorption isotherms of *Sp* CBP21 and *Sp* CBP50, towards α- and β-chitin, were obtained as described by Vaaje-Kolstad et al., [7] with minor modification. Varied concentration of an *Sp* CBP, up to 10.0 µM, was incubated with α-/β-chitin for different saturation periods: 12 h for α-chitin and 6 h for β-chitin with *Sp* CBP21, and 12 h for both α- and β-chitin with *Sp* CBP50.

### Soluble substrate binding specificity

Binding of Sp CBPs to soluble polysaccharides (glycol chitin, laminarin and CM cellulose) was evaluated by affinity electrophoresis as described by Hardt and Laine [24] with slight modifications. Proteins (10 µg of *Sp* CBPs and non-interacting BSA) were electrophoresed in 8.0% polyacrylamide gels impregnated with substrates (glycol chitin or laminarin or CM-cellulose) under non-denaturing conditions at 4°C. The gels were visualized by staining with Coomassie blue G-250.

### Sequence alignment for *Sp* CBPs and homology modelling of *Sp* CBP21and *Sp* CBP50

All three *Sp* CBPs from *S. proteamaculans* were aligned with *Sm* CBP21 using clustalw2 (www.ebi.ac.uk/Tools/msa/clustalw2/). A 3D structure models of *Sp* CBP21 and *Sp* CBP50 were generated using the template structure of *Sm* CBP21 (PDM ID: 2BEM) by Modeller9v8 (http://www.salilab.org/modeller/). About 40 models and corresponding Ramachandran plots were generated for each protein to check the protein structure quality using PROCHECK. The figures were prepared using PyMOL (http://www.pymol.org/).

Synergistic effect of *Sp* CBP21 and *Sp* CBP50 with *Sp* chitinases in chitin degradation.

Chitin degradation assay was performed as described by Vaaje-Kolstad et al., [19] with few modifications. A standard 1 mL reaction mixture containing 0.25 mg/mL of chitin substrates (α- or β-chitin), 1.0 µM of *Sp* chitinase (unless stated otherwise) [*Sp chiA*, *Sp chiB*, *Sp chiC* and *Sp chiD* (GenBank accession no. ABV39247.1, ABV40327.1, ABV42574.1 and ABV41826.1)] were amplified using gene specific primers (Table 2) and cloned, expressed, and purified similarly like *Sp cbps* (data not shown) and 1.0 mM reduced glutathione. Reaction mixtures were incubated in triplicates at 37°C for 7 days at 1000 rpm in a thermomixer. After every 24 h, 100 µL of reaction mixture was transferred and mixed with 100 µL of 0.02 N NaOH was added to stop the reaction and stored at −20°C until products quantification. Products were quantified by standard chitinase assay as described by Neeraja et al. (2010a, 22).

## Supporting Information

Figure S1 **Ni-NTA agarose purification of** ***Sp*** **CBPs.** Recombinant *Sp* CBP21, *Sp* CBP28 and *Sp* CBP50 were purified using Ni-NTA agarose column chromatography. Elution buffer containing 250 mM imidazole was used to elute *Sp* CBPs from the column and loaded on 12% SDS-PAGE followed by staining with Coomassie brilliant blue G-250. Lane 1: Protein standards size in kDa indicated to the left, lane 2–4: Purified *Sp* CBP21, *Sp* CBP28 and *Sp* CBP50, respectively.
(DOCX)

Figure S2 **Binding of** ***Sp*** **CBPs towards soluble polymeric substrates.** Affinity non-denaturing gel electrophoresis was performed at 4°C by preparing 8% polyacrylamide gels. Ten micrograms of Sp CBPs and BSA were electrophoresed without (A), or with 0.1% (w/v) substrates glycol chitin (B), laminarin (C), and CM-cellulose (D). Proteins were visualized by Coomassie blue G-250 staining after electrophoresis. Lane 1: BSA, lane 2–4: *Sp* CBP21, *Sp* CBP28 and *Sp* CBP50.
(DOCX)

Figure S3 **Dose-response effects for** ***Sp*** **CBP21 and** ***Sp*** **CBP50 in degradation of β-chitin.** Reaction mixture containing 0.25 mg/mL of chitin substrates (α-/β-chitin), 1.0 µM *Sp* ChiD incubated with different concentrations of *Sp* CBP21/*Sp* CBP50 (0.05–0.40 µM) as indicated in 50 mM sodium phosphate buffer pH 7.0. After incubation at 37°C for 24 h at 1000 rpm, 100 µL of reaction mixture was transferred. To this 100 µL of 0.02N NaOH was added to stop the reaction and stored at −20°C until products quantification by standard reducing end assay. Vertical bars represent standard deviation of triplicate experiments. (A and B) degradation of α- and β-chitin by *Sp* ChiD in the presence/absence of *Sp* CBP21 and reduced glutathione (RG), (C and D) degradation of α- and β-chitin by *Sp* ChiD in the presence/absence of *Sp* CBP50 and reduced glutathione (RG). *Sp* CBP21+RG or *Sp* CBP50+RG: *Sp* CBP21/*Sp* CBP50 with reduced glutathione, *Sp* CBP21+ or *Sp* CBP50+: only *Sp* CBP21/*Sp* CBP50 without reduced gluthathione, *Sp* CBP21- or *Sp* CBP50 -: without *Sp* CBP21/ *Sp* CBP50 and reduced glutathione.
(DOCX)
