# Supplementary material for: Chitin Binding Proteins Act Synergistically with Chitinases in Serratia proteamaculans 568
Source: PLoS One. 2012 May 9;7(5):e36714. doi: 10.1371/journal.pone.0036714 (PMC3348882; doi:10.1371/journal.pone.0036714)
Supplement: Figure S1 — Ni-NTA agarose purification of Sp CBPs. Recombinant Sp CBP21, Sp CBP28 and Sp CBP50 were purified using Ni-NTA agarose column chromatography. Elution buffer containing 250 mM imidazole was used to elute Sp CBPs from the column and loaded on 12% SDS-PAGE followed by staining with Coomassie brilliant blue G-250. Lane 1: Protein standards size in kDa indicated to the left, lane 2–4: Purified Sp CBP21, Sp CBP28 and Sp CBP50, respectively. (DOCX) [file pone.0036714.s001.docx]

**Figure S1.**


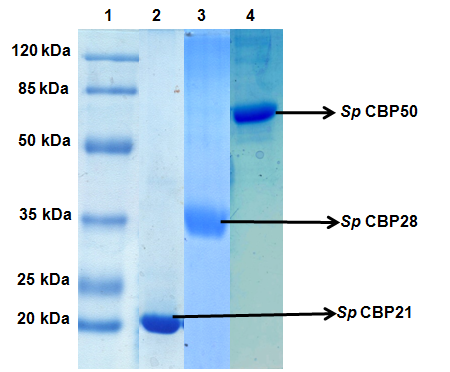


**Ni-NTA agarose purification of *Sp* CBPs.** Recombinant *Sp* CBP21, *Sp* CBP28 and *Sp* CBP50 were purified using Ni-NTA agarose column chromatography. Elution buffer containing 250 mM imidazole was used to elute *Sp* CBPs from the column and loaded on 12% SDS-PAGE followed by staining with Coomassie brilliant blue G-250. Lane 1: Protein standards size in kDa indicated to the left, lane 2- 4: Purified *Sp* CBP21, *Sp* CBP28 and *Sp* CBP50, respectively.
