# Supplementary material for: Chitin Binding Proteins Act Synergistically with Chitinases in Serratia proteamaculans 568
Source: PLoS One. 2012 May 9;7(5):e36714. doi: 10.1371/journal.pone.0036714 (PMC3348882; doi:10.1371/journal.pone.0036714)
Supplement: Figure S2 — Binding of Sp CBPs towards soluble polymeric substrates. Affinity non-denaturing gel electrophoresis was performed at 4°C by preparing 8% polyacrylamide gels. Ten micrograms of Sp CBPs and BSA were electrophoresed without (A), or with 0.1% (w/v) substrates glycol chitin (B), laminarin (C), and CM-cellulose (D). Proteins were visualized by Coomassie blue G-250 staining after electrophoresis. Lane 1: BSA, lane 2–4: Sp CBP21, Sp CBP28 and Sp CBP50. (DOCX) [file pone.0036714.s002.docx]

**Figure S2.**

**D**

**C**

**B**

**A**

1 2 3 4

1 2 3 4

1 2 3 4

1 2 3 4


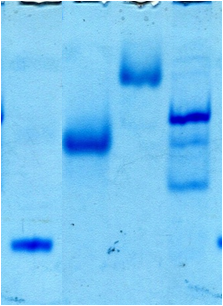

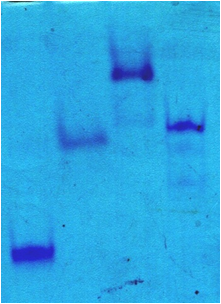

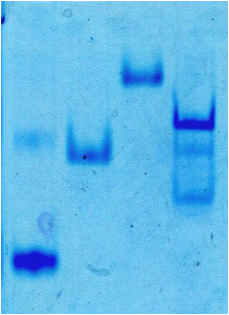

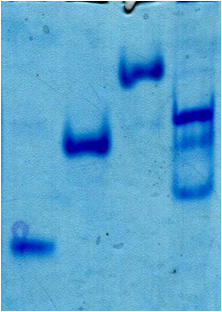


**Binding of *Sp* CBPs towards soluble polymeric substrates.** Aﬃnity non-denaturing gel electrophoresis was performed at 4°C by preparing 8% polyacrylamide gels. Ten micrograms of *Sp* CBPs and BSA were electrophoresed without (A), or with 0.1% (w/v) substrates glycol chitin (B), laminarin (C), and CM-cellulose (D). Proteins were visualized by Coomassie blue G-250 staining after electrophoresis. Lane 1: BSA, lane 2-4: *Sp* CBP21, *Sp* CBP28 and *Sp* CBP50.
