# Supplementary material for: Chitin Binding Proteins Act Synergistically with Chitinases in Serratia proteamaculans 568
Source: PLoS One. 2012 May 9;7(5):e36714. doi: 10.1371/journal.pone.0036714 (PMC3348882; doi:10.1371/journal.pone.0036714)
Supplement: Figure S3 — Dose-response effects for Sp CBP21 and Sp CBP50 in degradation of β-chitin. Reaction mixture containing 0.25 mg/mL of chitin substrates (α-/β-chitin), 1.0 µM Sp ChiD incubated with different concentrations of Sp CBP21/Sp CBP50 (0.05–0.40 µM) as indicated in 50 mM sodium phosphate buffer pH 7.0. After incubation at 37°C for 24 h at 1000 rpm, 100 µL of reaction mixture was transferred. To this 100 µL of 0.02N NaOH was added to stop the reaction and stored at −20°C until products quantification by standard reducing end assay. Vertical bars represent standard deviation of triplicate experiments. (A and B) degradation of α- and β-chitin by Sp ChiD in the presence/absence of Sp CBP21 and reduced glutathione (RG), (C and D) degradation of α- and β-chitin by Sp ChiD in the presence/absence of Sp CBP50 and reduced glutathione (RG). Sp CBP21+RG or Sp CBP50+RG: Sp CBP21/Sp CBP50 with reduced glutathione, Sp CBP21+ or Sp CBP50+: only Sp CBP21/Sp CBP50 without reduced gluthathione, Sp CBP21- or Sp CBP50 -: without Sp CBP21/ Sp CBP50 and reduced glutathione. (DOCX) [file pone.0036714.s003.docx]

**Figure S3.**

**A**

**B**

**C**

**D**

**Dose-response effects for *Sp* CBP21 and *Sp* CBP50 in degradation of β-chitin.** Reaction mixture containing 0.25 mg/mL of chitin substrates (α-/β-chitin), 1.0 µM *Sp* ChiD incubated with different concentrations of *Sp* CBP21/*Sp* CBP50 (0.05-0.40 µM) as indicated in 50 mM sodium phosphate buffer pH 7.0. After incubation at 37°C for 24 h at 1000 rpm, 100 µL of reaction mixture was transferred. To this 100 µL of 0.02N NaOH was added to stop the reaction and stored at -20°C until products quantification by standard reducing end assay. Vertical bars represent standard deviation of triplicate experiments. (A and B) degradation of α- and β-chitin by *Sp* ChiD in the presence/absence of *Sp* CBP21 and reduced glutathione (RG), (C and D) degradation of α- and β-chitin by *Sp* ChiD in the presence/absence of *Sp* CBP50 and reduced glutathione (RG).  *Sp* CBP21+RG or *Sp* CBP50+RG: *Sp* CBP21/*Sp* CBP50 with reduced glutathione, *Sp* CBP21+ or *Sp* CBP50+: only *Sp* CBP21/*Sp* CBP50 without reduced gluthathione, *Sp* CBP21- or *Sp* CBP50 -: without *Sp* CBP21/ *Sp* CBP50 and reduced glutathione.
